# First chromosome scale genomes of ithomiine butterflies (Nymphalidae: Ithomiini): Comparative models for mimicry genetic studies

**DOI:** 10.1111/1755-0998.13749

**Published:** 2023-01-27

**Authors:** Jérémy Gauthier, Joana Meier, Fabrice Legeai, Melanie McClure, Annabel Whibley, Anthony Bretaudeau, Hélène Boulain, Hugues Parrinello, Sam T. Mugford, Richard Durbin, Chenxi Zhou, Shane McCarthy, Christopher W. Wheat, Florence Piron-Prunier, Christelle Monsempes, Marie-Christine François, Paul Jay, Camille Noûs, Emma Persyn, Emmanuelle Jacquin-Joly, Camille Meslin, Nicolas Montagné, Claire Lemaitre, Marianne Elias

**Affiliations:** 1 Natural History Museum of Geneva, Geneva, Switzerland; 2 Department of Zoology, University of Cambridge, Cambridge, UK; 3 BIPAA, IGEPP, INRAE, Institut Agro, Univ Rennes, Rennes, France; 4 Univ Rennes, Inria, CNRS, IRISA, Rennes, France; 5 Institut Systématique Évolution Biodiversité (ISYEB), Centre National de la Recherche Scientifique, MNHN, EPHE, Sorbonne Université, Université des Antilles, Paris, France; 6 Laboratoire Écologie, Évolution, Interactions des Systèmes Amazoniens (LEEISA), Université de Guyane, CNRS, IFREMER, Cayenne, France; 7 School of Biological Sciences, University of Auckland, Auckland, New Zealand; 8 Department of Ecology and Evolution, University of Lausanne, Lausanne, Switzerland; 9 MGX-Montpellier GenomiX, Univ. Montpellier, CNRS, INSERM, Montpellier, France; 10 Department of Crop Genetics, John Innes Centre, Norwich Research Park, Norwich, UK; 11 Department of Genetics, University of Cambridge, Cambridge, UK; 12 Tree of Life Programme, Wellcome Sanger Institute, Hinxton, UK; 13 Department of Zoology, Stockholm University, Stockholm, Sweden; 14 Institute of Ecology and Environmental Sciences of Paris, Sorbonne Université, INRAE, CNRS, IRD, UPEC, Université de Paris, Paris, France; 15 Ecologie Systématique Evolution, Bâtiment 360, CNRS, AgroParisTech, Université Paris-Saclay, Orsay, France; 16 Laboratoire Cogitamus, Paris, France; 17 CIRAD, UMR PVBMT, St Pierre, France

**Keywords:** chromosome-level genome, Hi-C, ithomiine butterflies, mimicry, olfaction

## Abstract

The ithomiine butterflies (Nymphalidae: Danainae) represent the largest known radiation of Müllerian mimetic butterflies. They dominate by number the mimetic butterfly communities, which include species such as the iconic neotropical *Heliconius* genus. Recent studies on the ecology and genetics of speciation in Ithomiini have suggested that sexual pheromones, colour pattern and perhaps hostplant could drive reproductive isolation. However, no reference genome was available for Ithomiini, which has hindered further exploration on the genetic architecture of these candidate traits, and more generally on the genomic patterns of divergence. Here, we generated high-quality, chromosome-scale genome assemblies for two *Melinaea* species, *M. marsaeus* and *M. menophilus*, and a draft genome of the species *Ithomia salapia*. We obtained genomes with a size ranging from 396 to 503 Mb across the three species and scaffold N50 of 40.5 and 23.2 Mb for the two chromosome-scale assemblies. Using collinearity analyses we identified massive rearrangements between the two closely related *Melinaea* species. An annotation of transposable elements and gene content was performed, as well as a specialist annotation to target chemosensory genes, which is crucial for host plant detection and mate recognition in mimetic species. A comparative genomic approach revealed independent gene expansions in ithomiines and particularly in gustatory receptor genes. These first three genomes of ithomiine mimetic butterflies constitute a valuable addition and a welcome comparison to existing biological models such as *Heliconius*, and will enable further understanding of the mechanisms of adaptation in butterflies.

## 1 Introduction

The butterfly tribe Ithomiini (Nymphalidae: Danainae), which comprises 393 species, represents the largest known radiation of Müllerian mimetic butterflies, whereby co-occurring chemically-defended species converge in wing colour pattern, which acts as a warning signal learned and avoided by predators (Muller, 1879; Sherratt, 2008). Ithomiine butterflies are endemic to the neotropics, where they numerically dominate butterfly communities in lowland and mountain forests up to 2500 m, and where they engage in mimetic interactions with many other Lepidoptera (Beccaloni, 1997).

As such, ithomiine butterflies have an important ecological relevance. It is thus no wonder that ithomiine species served as examples in Bates’ (Bates, 1862) and Müller’s (Muller, 1879) original descriptions of Batesian (where palatable prey mimic distasteful ones) and Müllerian mimicry, respectively. Ithomiine butterflies are also remarkable in that many species have the unusual characteristic of harbouring partially transparent or translucent wings (McClure, Clerc, et al., 2019; Pinna et al., 2021). Mimetic butterflies have long attracted speciation research, as they usually feature assortative mating for wing colour patterns (e.g., Jiggins et al., 2001), combined with selection against hybrids between forms with different colour patterns (e.g., Merrill et al., 2014), because such hybrids typically harbour intermediate, nonmimetic colour patterns. The iconic genus *Heliconius* has been the focus of multiple such speciation studies, using both experimental (Jiggins et al., 2001; Merrill et al., 2014) and genomic (Martin et al., 2013; Merrill et al., 2019; Nadeau et al., 2012) approaches.

While colour pattern is believed to be a strong driver of diversification of mimetic butterflies (Kozak et al., 2015), including, possibly, Ithomiini (Jiggins et al., 2006), chemosensory communication may also be involved in speciation. Selection for similarity on a mating cue among co-occurring species is likely to result in reproductive interference (Boussens-Dumon & Llaurens, 2021; Estrada & Jiggins, 2008), raising the question of alternative mate recognition cues. Chemical signals such as sex pheromones have been suggested to a play a role in reproductive isolation in mimetic butterflies (Darragh et al., 2020; González-Rojas et al., 2020), particularly among co-mimetic species (Mérot et al., 2015). In ithomiine butterflies putative sexual pheromones have long been studied (Schulz et al., 2004), and have been shown to diverge between closely related taxa (Mann et al., 2020; McClure, Mahrouche, et al., 2019; Stamm et al., 2019), suggesting a possible role in reproductive isolation (McClure, Clerc, et al., 2019). More broadly, butterflies are phytophagous during the larval stage, and hostplant adaptation, mediated by chemical communication, has been hypothesized to be a major driver of speciation (Ehrlich & Raven, 1964; Jousselin & Elias, 2019). In Ithomiini, where butterfly-plant interaction tends to be very specific (Willmott & Mallet, 2004), divergent selection on hostplant has been documented (e.g., McClure & Elias, 2016b). Chemosensory and associated genes (i.e., all genes involved in chemical communication) thus represent particularly relevant targets for the study of speciation in mimetic butterflies. In butterflies, the detection of chemical signals is mainly performed by three types of membrane receptors named odorant receptors (ORs), gustatory receptors (GRs) and ionotropic receptors (IRs) and two secreted proteins families, the odorant-binding proteins (OBPs) and the chemosensory proteins (CSPs) (Pelosi et al., 2006; Robertson, 2019). The role of specific lineages of the OR gene family in the detection of volatile sex pheromones has been characterized in moths (Montagné et al., 2021). However, little is known of the molecular bases of pheromone detection in butterflies (Eyres et al., 2016; van Schooten et al., 2020). In Ithomiini, only one recent study addressed chemosensory genes, and found that one OR was differentially expressed between two subspecies of *Melinaea marsaeus* (Piron-Prunier et al., 2021), suggesting a possible role of chemical communication in mate choice.

Likewise, in contrast to *Heliconius*, little is known on the over-all genomic patterns of speciation in Ithomiini. Two studies, one using microsatellites and the other relying on reduced-complexity genomic data, revealed a range of levels of genetic differentiation among subspecies in five ithomiine species (Gauthier et al., 2020; McClure, Mahrouche, et al., 2019), calling for more in depth studies of population genetic structure and patterns of gene flow.

Despite these needs, research on speciation in Ithomiini is hindered by the lack of reference genomes. The paucity of genomic resources for Ithomiini is surprising, given their ecological and historical importance. The closest reference genome is that of the monarch butterfly, *Danaus plexippus* (Gu et al., 2019; Zhan et al., 2011), which belongs to the nymphalid tribe Danaini and that diverged from the Ithomiini tribe circa 42 million years ago (Chazot et al., 2019).

**Figure 1:**
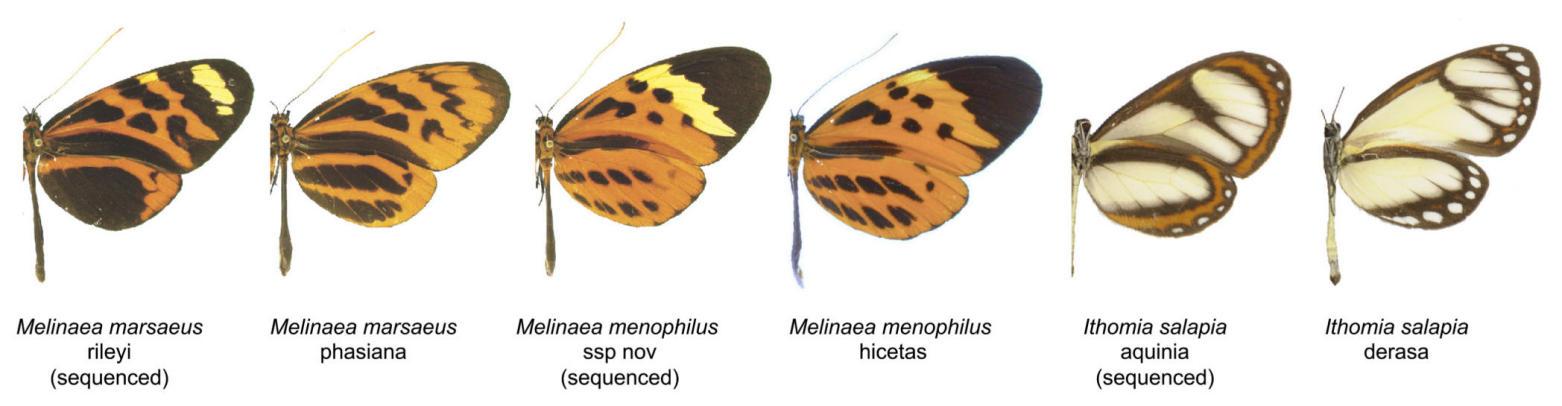
*Melinaea marsaeus, Melinaea menophilus, Ithomia salapia* and wing pattern variation between subspecies of each of these species (source Joron et al., 2006 and photograph credits Céline Houssin)

Here we present the first genomes of three Ithomiini species, *Ithomia salapia* (subspecies *aquinia*), *Melinaea marsaeus* (subspecies *rileyi*) and *Melinaea menophilus* (subspecies ssp *nov*). *Ithomia salapia* is a typical “clearwing” ithomiine butterfly, in that it shows transparent or translucent wings (Figure 1). Subspecies of *I. salapia* belong to large mimicry rings that include ithomiine and non ithomiine species (Beccaloni, 1997; Willmott et al., 2017). The genus *Ithomia* belongs to the Ithomiine “core-group”, a clade that encompasses 80% of the species of the tribe and that underwent steady diversification in the Central Andes during the Miocene before colonizing other neotropical regions (Chazot et al., 2019). A recent population genetic study in a suture zone showed that gene flow between subspecies of *I. salapia* was highly reduced, suggesting incipient speciation (Gauthier et al., 2020). The genus *Melinaea* (Figure 1) belongs to a basal Amazonian lineage that probably experienced high extinction rates during the Miocene before diversifying at a higher pace during the last couple of million years (Chazot et al., 2019). *Melinaea* species engage in mimetic interactions with multiple other Lepidoptera, including species from the tribe Heliconiini (Beccaloni, 1997). Also, and contrasting with *I. salapia*, genetic studies based on microsatellite and coding sequences found an exceptionally low level of divergence among *Melinaea* subspecies and even species (Chazot et al., 2019; Dasmahapatra et al., 2010; McClure, Mahrouche, et al., 2019), which may indicate recent diversification or extensive gene flow. Another intriguing feature in the genus *Melinaea* is the high karyotypic lability, with multiple events of chromosomal fusion recorded between two closely related subspecies (Brown Jr et al., 2004; McClure et al., 2017).

Because the genomes of these three species are large and highly heterozygous, it has been necessary to test and combine different sequencing methods. The genomes of *M. marsaeus* and *M. menophilus* presented here were assembled combining PacBio HiFi, 10x and HiC, which allowed us to assemble genomes at the chromosome level. The *I. salapia* genome, obtained with 10x sequencing, is more fragmented and can be considered as a draft genome. For each of the genomes we generated gene annotations using a pipeline that incorporated transcriptomic data and manually annotated the chemosensory gene families, as these families are usually badly predicted by automatic annotations.

## 2 Materials and Methods

### 2.1 Sample collection, DNA extraction, library construction and sequencing

Females of *I. salapia aquinia* were collected in Urahuasha (6°27’ S, 76°20 W, San Martin, Peru) and kept in captivity, where they were presented with potted *Witheringia solanacea* for egg-laying. Females of *M. marsaeus rileyi* and *M. menophilus ssp nov* were collected in Micaela (5°56’ S, 76°14’ W, Loreto province, Peru), and Urahuasha, respectively, and kept in captivity in Tarapoto (San Martin, Peru), where they were presented with potted *Juanulloa parasitica* on which they laid eggs. Larvae of all species were reared on their host plants until pupation, and pupae were preserved in empty plastic vials at −80°C until extraction.

For the genomes of *M. marsaeus* (ilMelMars1.1) and *M. menophilus* (ilMelMeno1.1), DNA extraction, library preparation and sequencing were performed by the Scientific Operations core at the Wellcome Sanger Institute. DNA was extracted from flash-frozen pupae of female butterflies with the Qiagen MagAttract HMW DNA kit. Pacific Biosciences (PacBio) HiFi libraries were sequenced on a PacBio SEQUEL II. 10x Genomics Chromium version 2 libraries and HiC Arima version 2.0 libraries were constructed according to the manufacturer’s instructions and sequenced on Illumina HiSeq X instruments.

The two individuals used for the genome of *I. salapia* were extracted following a protocol adapted from (Mayjonade et al., 2016). Samples were snap frozen alive in liquid nitrogen and conserved at −80°C. DNA was extracted from the whole butterfly bodies with the exception of the head. Butterflies were ground in a frozen mortar with liquid nitrogen, 150 mg of tissue powder was mixed with 900 μl of preheated buffer and 6 μl of RNaseA. Tubes were incubated for 120 min at 50°C for lysis, and then at −10°C for 10 min, with the addition of 300 μl of potassium acetate for the precipitation. One volume of binding buffer was added with 100 μl of Serapure beads solution. Three washing cycles were used and DNA was resuspended in 100 μl of EB buffer. Library construction including adaptor ligation and size selection were performed according to the manufacturer’s instructions. The two 10x Chromium Genome Library libraries were sequenced on one lane of the HiSeq 2500 with a 250PE-RR read metric.

### 2.2 Transcriptomic data

For *M. marsaeus* and *I. salapia* transcriptomic data were generated from various tissues including (abdomen, thorax, head) and developmental stages (adult, pupae and two larval stages) (detailed in Table 1) to maximize transcript diversity. In addition, targeted tissues from pupal wing discs and antennae in *M. marsaeus* were used (Piron-Prunier et al., 2021). Tissue samples were homogenized in 600 μl of RLT buffer with TissueLyser (Qiagen). Total RNA was then extracted according to the manufacturer’s protocol (RNeasy Mini kit, Qiagen) and eluted in 30 μl of RNase-free water. To avoid genomic contamination, RNase-free DNase treatment (Qiagen) was performed during RNA extraction. The quality of the isolated RNA was checked on 0.8% agarose gel for the presence of 28 S and 18 S bands. The quality and quantity of RNA was further analysed using Qubit 2.0 fluorometer (Invitrogen) and RNA integrity was confirmed using an Agilent Bioanalyser 2100 (Agilent Technologies). Libraries were sequenced with Illumina HiSeq 2500 platform.

### 2.3 Genome size and heterozygosity estimation using k-mers approaches

Genome characteristics, genome size, heterozygosity, were estimated on each data set of raw reads using k-mer spectrum distribution analysis. K-mer distribution were estimated using JELLYFISH version 2.2.10 (Marçais & Kingsford, 2011) and a k-mer size of 31. GENOMESCOPE2 (Ranallo-Benavidez et al., 2020) was used to estimate genome characteristics and generate plots (Figure S1).

### 2.4 Genome assembly

For *M. marsaeus* and *M. menophilus*, the assembly process included the following sequence of steps: initial PacBio assembly generation with Hifiasm version 0.15.1 (Cheng et al., 2021), retained haplotig separation with purge_dups version 1.2.3 (Guan et al., 2020), short-read polishing using FreeBayes version 1.3.1-called variants (Garrison & Marth, 2012) from 10x Genomics Chromium reads aligned with LongRanger version 2.2.2 (https://github.com/10XGenomics/longranger), and Hi-C based scaffolding with SALSA2 version 2.2 (Ghurye et al., 2019) using Hi-C contact map (Figure S2). The mitochondrial genome was assembled using MitoHifi version 2 (https://github.com/marcelauliano/MitoHiFi). Finally, the assemblies were analysed and manually improved using rapid curation (Howe et al., 2021). Chromosome-scale scaffolds confirmed by the Hi-C data have been named in order of size. Genome completeness was assessed with BUSCO version 5 (Manni et al., 2021) using the “genome” mode with the lepidoptera_odb10 orthologue data set composed of 5286 orthologous genes. BUSCO genes were also used to identify the Z chromosomes in both species. The putative Z chromosomes also showed reduced read coverage in both species, supporting that they are Z chromosomes of females. In *M. menophilus* a second chromosome with reduced coverage, Hi-C links to the Z chromosome and very small size (2.99 Mbp) was assigned as putative W chromosome. For *I. salapia*, all 10x libraries of the two samples were first assembled separately with Supernova version 2.1.1 (Visendi, 2022) and then combined with Ragout using one genome as reference and the other one as target (Kolmogorov et al., 2018). Base accuracy (QV) was estimated using a k-mer size of 21 with Merqury (Rhie et al., 2020).

### 2.5 Synteny

Synteny between *M. marsaeus* and *M. menophilus* genomes was investigated using the positions of the complete nonduplicated BUSCO genes. Using a custom-made R script, we merged the BUSCO gene position files and plotted them against each other.

### 2.6 Gene prediction, automated and functional annotations

The transposable element annotation was realized using RepeatMasker (Tarailo-Graovac & Chen, 2009). This annotation was exported into GFF3 files and used as a mask for gene annotation. Later, repeat masking with de novo repeat discovery, automated curation and filtering was performed using the EarlGrey pipeline (version 1.2) (Baril et al., 2021) with default settings in combination with the *Arthropoda* library from the Dfam database (version 3.5) (Storer et al., 2021). The automated gene prediction and annotation was done using MAKER (Cantarel et al., 2008) integrating different features based on (i) the mapping of Lepidoptera proteins from LepBase (Challi et al., 2016), (ii) the transcriptomes of each species generated by the assembly of RNA-Seq data with Trinity 2.8.4 (Haas et al., 2013) and (iii) ab initio genes predictions using Augustus (Hoff & Stanke, 2019). Reliable gene predictions were extracted according to annotation edit distance (AED) ≤0.2 or a minimum coverage of 1000 from RNAseq data mapping after optimization using BUSCO statistics. Annotation completeness was assessed with BUSCO version 5 (Manni et al., 2021) using the “protein” mode with the lepidoptera_odb10 ortholog data set composed of 5286 orthologous genes. The functional annotation was performed using blastp from BLAST+ version 2.5.0 (Camacho et al., 2009) to compare predicted proteins in each genome to the NCBI nonredundant database. The 10 best hits below an e-value of 1 e-08 without complexity masking were conserved. Interproscan (Jones et al., 2014) was used to analyse protein sequences seeking for known protein domains in the different databases available in Interproscan. Finally, we used Blast2GO (Conesa et al., 2005) to associate a protein with a gene ontology (GO) group.

### 2.7 Orthologue analyses

Orthologous genes between annotated genes in each species and the seven outgroups (*Pieris napi, Bicyclus anynana, Junonia coenia, Melitaea cinxia, Heliconius erato, Heliconius melpomene* and *Danaus plexippus*) were identified using OrthoFinder version 2.5.2 (Emms & Kelly, 2015). Single copy orthologue proteins were extracted, aligned using MAFFT version 7.01775 and concatenated using AMAS (Borowiec, 2016). The species phylogeny was performed on this alignment composed of 996 orthologues for a length of 647 kb using PhyML (Anisimova & Gascuel, 2006) including a branch support estimation with 1000 bootstrap iterations.

### 2.8 Manual annotation of chemosensory genes

For each of the chemosensory gene family, that is, odorant receptors (ORs), the gustatory receptors (GRs), the variant ionotropic receptors (IRs), the odorant-binding proteins (OBPs) and the chemosensory proteins (CSPs), amino acid sequences previously identified from the genomes of *D. plexippus, H. melpomene, S. frugiperda* and *B. mori* (Briscoe et al., 2013; Gouin et al., 2017; Guo et al., 2017; Heliconius Genome Consortium, 2012; Liu et al., 2018; Meslin et al., 2022; Vogt et al., 2015; Zhan et al., 2011) as well as from the transcriptome of *M. marsaeus* (Piron-Prunier et al., 2021) were used as queries in a tBLASTn search against genome assemblies of the three species (e-value threshold 0.001), in order to identify scaffolds containing the genes to annotate. Query amino acid sequences were then aligned on these scaffolds with Exonerate (Slater & Birney, 2005) to identify precise intron-exon boundaries and create gene models. These models were visualized using Integrated Genomics Viewer version 2.11.9 (Robinson et al., 2011), and badly predicted models were eliminated from the final sequence data sets. Nucleotide and amino acid sequences were extracted with GffRead (Pertea & Pertea, 2020). To create CSP and GR trees, amino acid sequences from Ithomiini were aligned with those of the above-mentioned species (except *S. frugiperda* GRs that were not included to limit the number of sequences) with MAFFT version 7 (Katoh et al., 2019). Maximum-likelihood phylogenies were built using PhyML 3.0 (Guindon et al., 2010) following model selection by SMS (Lefort et al., 2017). Branch support was estimated via SH-like approximate likelihood-ratio test (Anisimova & Gascuel, 2006).

## 3 Results and Discussion

### 3.1 Sequencing strategy comparison

In order to obtain a high-quality reference genome for *M. marsaeus*, we combined deeper PacBio sequencing using the new HiFi technology with low error rates, 10x sequencing and HiC data (Table 1). The use of a HiC approach, which enabled us to organize the scaffolds at the chromosome level, was particularly successful as it resulted in a final genome of 503 Mb composed of 22 scaffolds and an N50 of 40.4 Mb (Table 2). The same strategy was used for the species *M. menophilus* and yielded similar quality results with a genome of 496 Mb composed of 28 scaffolds and an N50 of 23.1 Mb (Table 2). For *I. salapia*, two 10x libraries were generated from two individuals and sequenced separately (Table 1). Largely due to the absence of HiC libraries and PacBio HiFi libraries, the genome obtained for this species is more fragmented than those of the two *Melinaea* species. The final assembly is composed of 23,973 scaffolds for a total of 395 Mb and an N50 of 1.4 Mb (Table 2). For *M. marsaeus*, the 22 scaffolds obtained could be grouped into 13 chromosomes, two sex chromosomes W and Z, the mitochondrion and six unplaced scaffolds. For *M. menophilus*, the 28 scaffolds were grouped into 20 chromosomes, two sex chromosomes W and Z, the mitochondrion and five unplaced scaffolds. The final number of chromosomes assembled matches the number of chromosomes identified by cytogenetic techniques in *M. menophilus*, that is, 2 n = 42 (Dutrillaux et al., 2022).

### 3.2 Genome size and heterozygosity estimation

For each of the three genomes, the size of the final assemblies is within, or slightly above, the range of the size estimates from k-mer approaches on the raw reads. For *M. marsaeus* the k-mer estimates range from 330 to 496 Mb (Table S1) and the assembled genome size is 503 Mb; for *M. menophilus* the k-mer estimates range from 357 to 527 Mb (Table S1) and the assembled genome size is 496 Mb; and finally for *I. salapia*, the k-mer size estimate range is 352 to 357 Mb and the assembled genome size is 395 Mb (Table 2, Table S1). These genome sizes are at the top of the distribution of genome sizes observed in the Danainae, ranging from 249 to 455 Mb, but are below those of the largest genomes observed in the Nymphalidae, such as *Polyura nepenthes* (Nymphalidae, Charaxinae) whose genome size is estimated at 925 Mb (Liu et al., 2020). When comparing 10x data, almost four times more heterozygosity is observed for *M. marsaeus* than for *M. menophilus* (Table S1). The levels of heterozygosity estimated using k-mer approaches show an heterogeneity between the different data sets but seem to show a fairly high level of heterozygosity (Table S1). This may be related to the demographic history of the populations and, for *M. marsaeus*, to the mechanisms of divergence and hybridisation that exist in the suture zone between the Andes and the Amazon. The populations of *M. marsaeus* around Tarapoto were found to be profoundly admixed in a previous study (McClure & Elias, 2016a). This high level of divergence between *M. marsaeus* populations and their hybridisation may explain the difficulty of assembly encountered during the first attempt to sequence this species.

The final assemblies show a high level of completeness, as testified by high BUSCO completeness using the “genome” mode (Seppey et al., 2019). For each of the genomes, including the more fragmented genome of *I. salapia*, more than 95% of 5286 single copy orthologues across Lepidoptera were recovered (Table 2).

**Figure 2:**
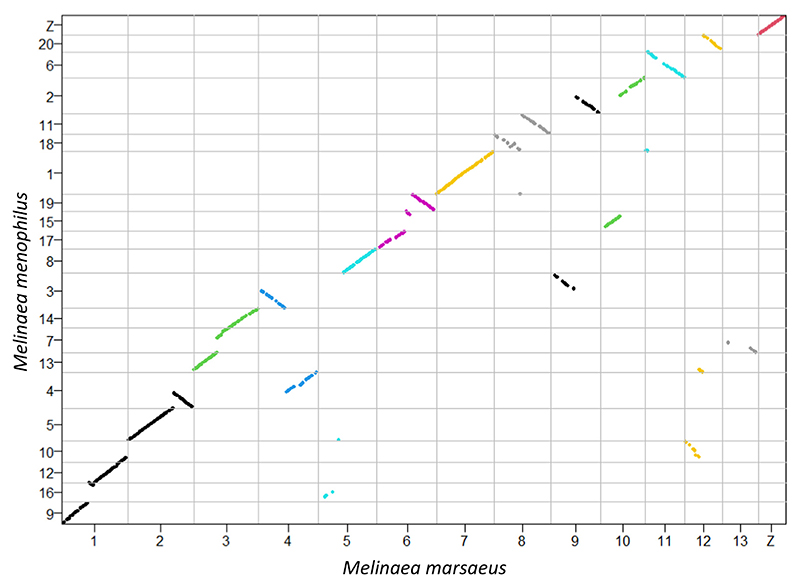
Low synteny between *M. marsaeus* and *M. menophilus* despite very recent splitting time. The positions of BUSCO genes mapping uniquely to both genomes are shown in the order of the *M. marsaeus* chromosomes. The colours reflect the different *M. marsaeus* chromosomes. A fully conserved chromosome would be reflected as a single diagonal line as in *M. marsaeus* chromosome 7, which corresponds to *M. menophilus* chromosome 1. Grey lines indicate chromosome ends

In contrast to the highly colinear genomes *Heliconius* butterflies, where most species have 21 chromosomes (Seixas et al., 2021), our closely related *Melinaea* species differ strongly in chromosome number (14 vs. 21) and show numerous massive rearrangements (Figure 2). The only two *M. marsaeus* chromosomes that fully correspond to a single *M. menophilus* chromosome, are chromosomes 7 (chr. 1 in *M. menophilus*) and the Z chromosome. The high variation in chromosome numbers in species in the genus *Melinaea* has already been observed by (Brown et al., 2004; Dutrillaux et al., 2022; McClure et al., 2017). Here we show that this variation could be the result of fusion and fission events.

### 3.3 Gene prediction and function annotation

Prior to the gene annotation step, an annotation of transposable and repeated elements was performed. To perform reliable gene annotation we took advantage of transcriptomic data. For *M. marsaeus*, we used assembled transcripts from a study on differential expression between two subspecies (Piron-Prunier et al., 2021), which included a reference transcriptome for that species across multiple stages (larval, pupal and imago) and transcriptomes of targeted tissues, namely pupal wing discs and antennae (Table 1). For *I. salapia*, we sequenced and assembled a reference transcriptome by sequencing transcripts from different tissues and different developmental stages (Table 1). Automated annotations combining transcriptomic data, known lepidopteran proteins and ab initio predictions annotated respectively 52,865 genes for *M. marsaeus*, 54,531 genes for *M. menophilus* and 32,213 for *I. salapia*. After the filtering of the reliable gene predictions, 18,670 genes were kept for *M. marsaeus*, 19,174 for *M. menophilus* and 18,283 for *I. salapia*. These genes have comparable characteristics in terms of gene size, number and sizes of exons and introns (Table 3). Like the genomes, these annotations and the predicted proteins have a high completeness level identified by BUSCO using the “protein” mode with more than 85.8% of the lepidopteran single copy orthologues recovered (Table 3).

Annotation of the repetitive elements of the genome, combining de novo and homology-based discovery approaches, revealed increased transposable element content with increasing genome size, with 14% total repeat content in *I. salapia* and 24% in the two *Melinaea* species (Figure S3). The differences could be linked to sequencing strategies. The complement of different element classes differed between the species and from the repeat content described in *Danaus* species, which themselves show considerable variation within the genus (Baril & Hayward, 2022). More specifically, the ithomiine genomes all exhibit increased DNA transposon, Rolling-circle and LINE and LTR retroelement content but decreased contributions of Penelope elements. SINE retroelements comprise nearly 3% of the genome assemblies in both Melinaea species but less than 0.2% of the *I. salapia* genome. Transposon landscape analysis supports recent transposon activity in all genomes, as indicated by the presence of several TE classifications with low genetic distance to their consensus sequences (Figure S2). Regarding the distribution at the chromosome level, the sex chromosomes have different concentrations of repeated elements than the autosomes. The Z chromosomes present only 14% of transposable elements for both species. Conversely, the W chromosomes have a much higher concentration than the autosomes, reaching 59.72% for *M. menophilus* and 73.62% for *M. marsaeus*. However, for both the Z and W chromosomes, the composition of the different families of transposable elements is substantially similar between the sex chromosomes and with the rest of the genome (Figure S3).

### 3.4 Comparison with key lepidopteran reference genomes

**Figure 3:**
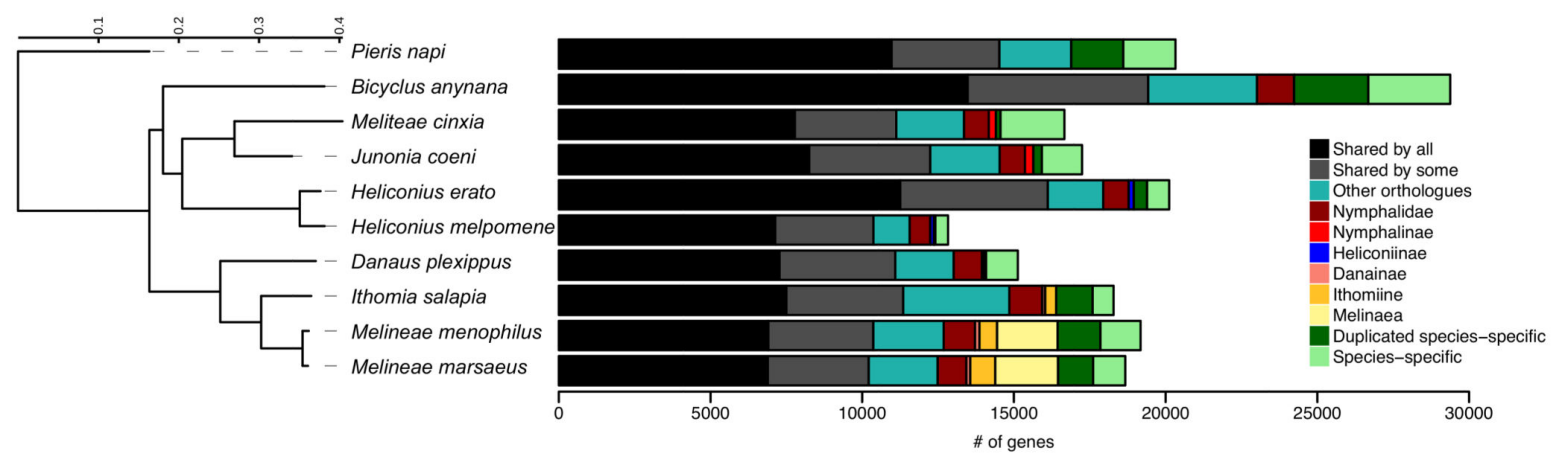
Phylogeny and orthologous gene numbers across 10 butterfly genomes. “Shared by some” represents orthologues shared by eight out of the 10 species and without phylogenetic signal

Orthologous genes for all annotated genes in the three focal species and seven outgroup butterfly species, including reference genomes such as *Danaus plexippus* (PRJNA564985), the species most closely related to the Ithomiini, and *Heliconius melpomene* (PRJEA71053), a species belonging to a large clade of mimetic butterflies, were identified using OrthoFinder version 1.1474 (Emms & Kelly, 2015). In total, 16,736 orthology groups were identified including 93.0% of all the analysed genes from the 10 species. Among them, 5792 orthogroups are shared by all species. Larger gene numbers were observed for the *Melinaea* species. Thus, a reduced proportion of genes are shared by the ithomiines, which represent 4.4 and 3.0% of the genes for *M. marsaeus* and *M. menophilus* respectively, and 2.0% of the genes for *I. salapia* (in light orange on Figure 3). Within *Melinaea*, a large proportion of genes are associated with the *Melinaeae* genus and shared between the two species, representing 11.1% of genes for *M. marsaeus* and 10.4% of genes for *M. menophilus* (in light yellow on Figure 3). Finally, we also observed a large proportion of species-specific genes, since they reach 11.9% (including 6.2% of duplicated species-specific) for *M. marsaeus* and 14.3% (including 7.4% of duplicated species-specific) for *M. menophilus* (in green on Figure 3).

### 3.5 Annotation of chemosensory genes

**Figure 4:**
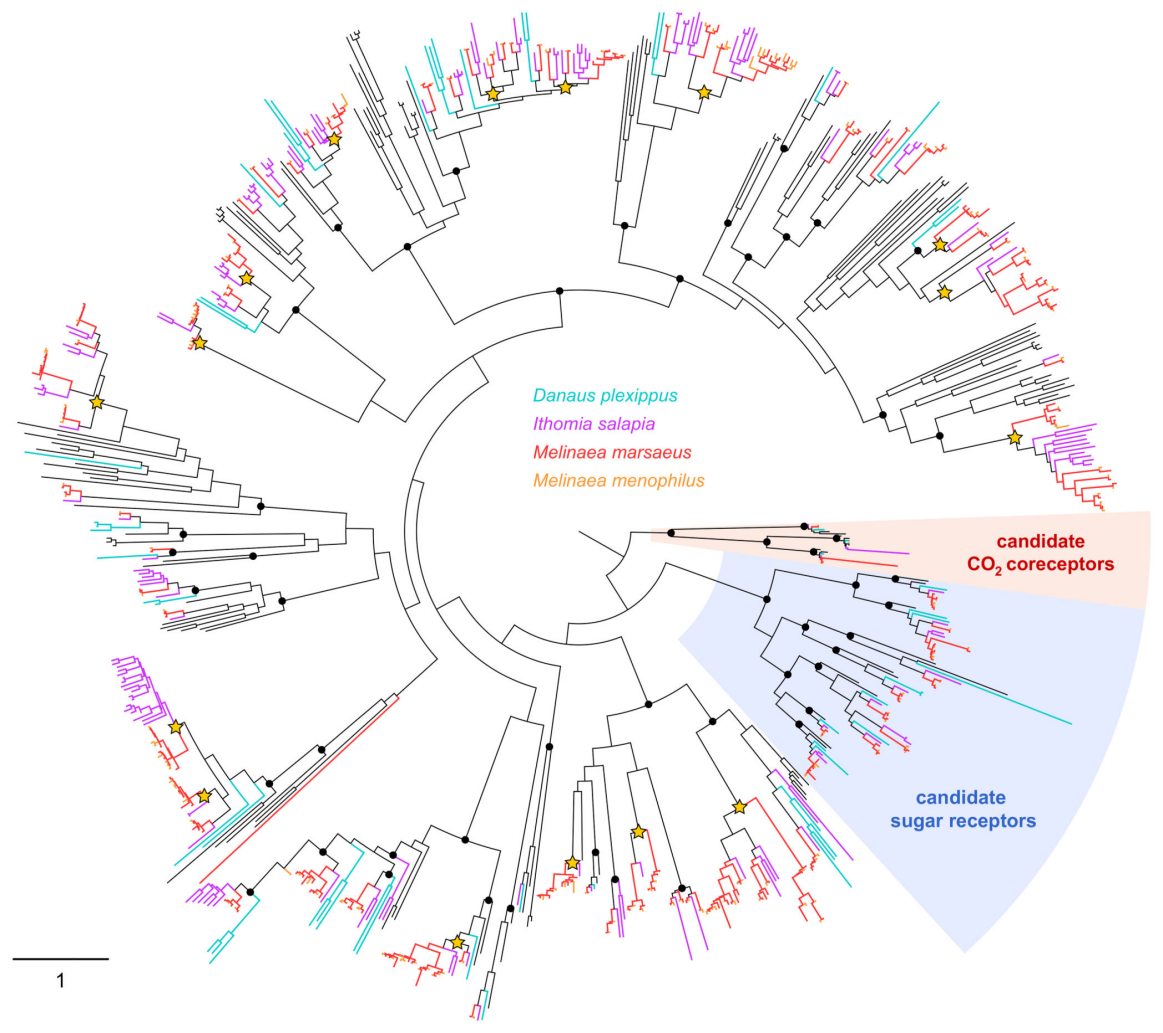
Maximum-likelihood phylogeny of lepidopteran GRs, built from amino acid sequences from *B. mori, H. melpomene, D. plexippus, I. salapia, M. marsaeus* and *M. menophilus*. Deep nodes highly supported by the likelihood-ratio test (aLRT >0.95) are indicated by black dots. Those that correspond to Ithomiini-specific large expansions (more than 10 genes) are shown with stars. The scale bar represents the expected number of amino acid substitutions per site

Chemosensory cues and signals are instrumental for butterflies as they are involved in host plant detection and in mate recognition. This is especially the case in mimetic butterflies, whereby the colour pattern may not provide an effective cue for mate recognition due to mimicry (Mérot et al., 2015). Detection of chemosensory cues and signals by the peripheral nervous system of insects is mainly governed by transmembrane receptors located at the membrane of olfactory or gustatory neurons, responsible for signal transduction upon ligand activation. In insects, such receptors belong to three multigene families: the odorant receptors (ORs), the gustatory receptors (GRs) and the variant ionotropic receptors (IRs). Depending on insect orders, the number of genes within each family can vary from a few dozens to several hundreds (Robertson, 2019). We annotated genes belonging to these families in the three Ithomiini genomes (Table 4). The number of OR genes varied from 62 in *M. menophilus* to 70 in *I. salapia*, which is similar to the number found in any other lepidopteran genome, including the closely related species *D. plexippus* (Montagné et al., 2021). The same holds true for IR genes whose numbers varied from 31 in *M. marsaeus* to 36 in *I. salapia*. By contrast, we annotated an unexpectedly large number of GR genes in the three species, up to more than 200 in *M. marsaeus*. This high number of genes compared with other Nymphalidae (including *D. plexippus*) results from extensive duplications in Ithomiini that occurred in several lineages of the GR phylogeny (Figure 4). So far, such expansions of GR repertoires in Lepidoptera have been documented only in the Noctuidae family, where it has been tentatively linked to polyphagy (Gouin et al., 2017; Meslin et al., 2022). It is interesting to note that somehow similar expansions also occurred independently in Ithomiini, which are not polyphagous but rather oligophagous species (McClure & Elias, 2016b; Willmott & Mallet, 2004).

**Figure 5:**
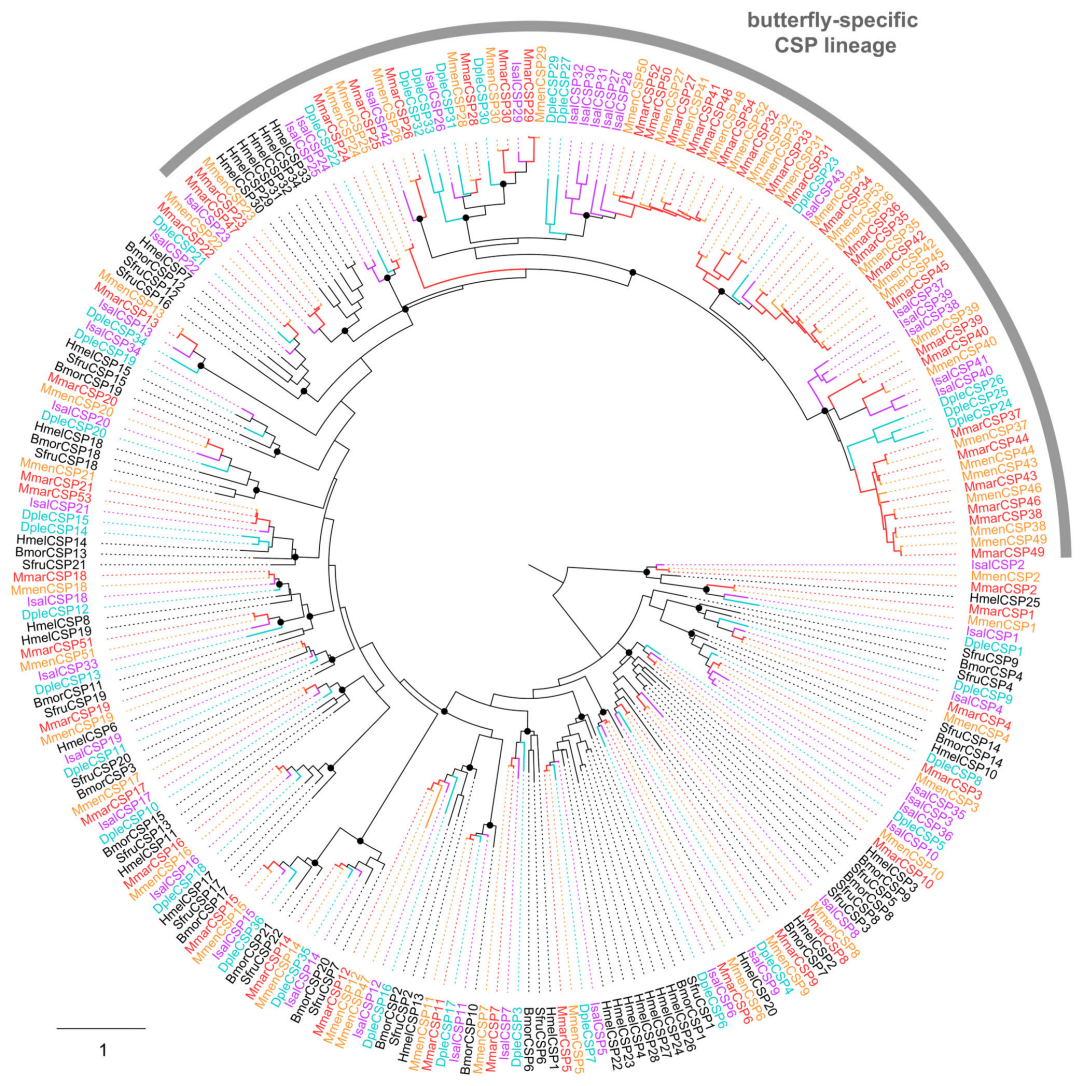
Maximum-likelihood phylogeny of lepidopteran CSPs, built from amino acid sequences from *B. mori* (Bmor), *S. frugiperda* (Sfru), *H. melpomene* (Hmel), *D. plexippus* (Dple), *I. salapia* (Isal), *M. marsaeus* (Mmar) and *M. menophilus* (Mmen). Deep nodes highly supported by the likelihood-ratio test (aLRT >0.95) are indicated by black dots. The scale bar represents the expected number of amino acid substitutions per site

Apart from transmembrane receptors, chemodetection in insects also relies on soluble proteins that can bind and transport semiochemicals in the aqueous lymph of olfactory and gustatory sensilla, so that they can reach the neurons. The genomes of Ithomiini contain 35 to 40 genes encoding odorant-binding proteins (OBPs), which is in the range of what has been observed in other Lepidoptera. On the other hand, the number of chemosensory proteins (CSPs) is exceptionally high in Ithomiini genomes, especially in both *Melinaea* species which have more than 50 CSP genes (Table 4). The phylogenetic analysis shows that all but one of the CSP lineages are highly conserved in Lepidoptera, whereas numerous gene duplications occurred in a butterfly-specific lineage (Figure 5). This expansion has been documented previously (Heliconius Genome Consortium, 2012) yet it is particularly spectacular in Ithomiini genomes, which contain up to 30 CSP genes (in *M. marsaeus*) versus 11 in *D. plexippus* and six in *H. melpomene*. This further confirms a previous observation made following the analysis of the *M. marsaeus* transcriptome (Piron-Prunier et al., 2021).

## 4 Conclusion

In this study we sequenced, de novo assembled, and annotated the genomes of three ithomiine species. We analysed their genomic features and performed genomic content comparison and orthologous gene identification with *D. plexippus*, which belongs to the same sub-family (Danainae), and various outgroups including two *Heliconius* species (Nymphalidae: Heliconiinae), a well-studied mimetic genus that includes species that are mimetic with *Melinaea*. Manual curation of chemosensory genes in the three genomes revealed unexpected expansions of GR genes, as has been previously observed only in polyphagous noctuids. These first genomes of ithomiine mimetic butterflies will be useful to further understand the mechanisms of adaptation and the genetic bases underpinning mimicry, and provide a welcome comparison to existing biological models of mimicry like the *Heliconius*.

## Supplementary Material

Appendix S1

## Figures and Tables

**Table 1:** Statistics of raw read data including sequencing strategy, read length, number of reads and total sequenced bases

Species	Sequencing strategy	Read length (bp)	No. reads	No. bases (Gb)
Genomic data				
*M. marsaeus*	PacBio HiFi	N50 = 11,228	2,593,875	26.81
	10x	151	146,203,982	22.07
	HiC	151	118,404,072	17.88
*M. menophilus*	PacBio HiFi	N50 = 11,996	2,275,183	25.20
	10x	151	118,378,976	17.87
	HiC	151	147,947,432	22.34
*I. salapia*	10x	150	163,421,078	24.51
	10x	150	155,015,880	23.25
**Species**	**Tissue**	**Read length (bp)**	**No. reads**	**No. bases (Gb)**
Transcriptomic data				
*M. marsaeus* *	Thorax	150	72,739,116	10.91
	Abdomen	150	67,825,765	10.17
	Head	150	76,024,296	11.40
	Pupae	150	79,408,138	11.91
	Fifth instar caterpillar	150	82,451,250	12.37
	Pupae wing disks	150	376,165,512	56.42
	Adult antenna	150	410,103,511	61.52
*I. salapia*	Thorax	150	181,315,214	27.20
	Abdomen	150	145,723,564	21.86
	Head	150	164,980,366	24.75
	Pupae	150	151,605,900	22.74
	Fifth instar caterpillar	150	149,181,784	22.38

* From Piron-Prunier et al. (2021).

**Table 2:** Assembly statistics and completeness evaluation

Assembly statistics	*M. marsaeus*	*M. menophilus*	*I. salapia*
No. scaffolds	*22*	28	23,973
N50 scaffold	40,461,556	23,164,123	1,472,785
L50 scaffold count	6	8	70
Mean scaffold size	22,886,664.64	17,716,406.50	16,515
Longest scaffold	46,264,634	41,164,108	15,188,582
%N	0,001	0,002	8.74
GC content (%)	31.7	31.7	30.99
Total length	503,506,622	496,059,382	395,915,617
**Base quality (QV)**			
PacBio HiFi	58.55	*58.87*	
10x	48.23	49.61	58.54
**BUSCO results on genomes**			
Complete and single-copy BUSCOs	96.8	98.1	95.0
Complete and duplicated BUSCOs	0.6	0.6	2.4
Fragmented BUSCOs	0.3	0.3	0.9
Missing BUSCOs	2.3	1.0	1.7

**Table 3:** Annotation statistics and predicted protein completeness evaluation

Gene statistics	*M. marsaeus*	*M. menophilus*	*I. salapia*
No. of raw genes	52,865	54,431	32,213
No. of filtered genes	18,670	19,174	18,283
Average gene length (bp)	5821.59	5779.93	5228.13
Median gene length (bp)	3877.00	3839.00	2889.00
Average exon per gene	6.78	6.68	6.09
Average exon length (bp)	257.52	262.14	239.17
Average intron per gene	5.78	5.68	5.09
Average intron length (bp)	643.28	650.95	659.45
% coding sequence	6.48	6.77	6.74
**BUSCO results on** **proteins**			
Complete and single-copy BUSCOs	85.8	87.5	88.4
Complete and duplicated BUSCOs	1.0	1.1	2.6
Fragmented BUSCOs	1.0	0.9	1.5
Missing BUSCOs	12.2	10.5	7.5

**Table 4:** Number of chemosensory genes annotated in different lepidopteran genomes

Species	OR	GR	IR	OBP	CSP
*M. marsaeus*	63	209	31	39	54
*M. menophilus*	62	187	34	35	53
*I. salapia*	70	167	36	40	43
*D. plexippus*	64	56	32	32	34
*H. melpomene*	66	73	33	51	33
*S. frugiperda*	69	234	42	50	22
*B. mori*	73	76	30	39	21

## Data Availability

Genome assemblies have been made available on the NCBI under GCA_918358865.1 accession number for *M. marsaeus*, GCA_918358695.1 for *M. menophilus* and JAPOND000000000 for I. *salapia*. Raw genomic data can be found under BioProjects PRJNA836751 (*M. marsaeus* v1), PRJEB48295 (*M. marsaeus* v2) and PRJEB48296 (*M. menophilus*). Raw transcriptomic data can be found under BioProjects PRJNA836751. The assembly and annotation pipelines including custom scripts have been made available in the Github repository https://github.com/JeremyLGauthier/scripts_Ithomiine_genomes.

## Open Research Badges

This article has earned an Open Data badge for making publicly available the digitally-shareable data necessary to reproduce the reported results. The data is available at https://github.com/JeremyLGauthier/scripts_Ithomiine_genomes.
