# Supplementary material for: First chromosome scale genomes of ithomiine butterflies (Nymphalidae: Ithomiini): Comparative models for mimicry genetic studies
Source: Mol Ecol Resour. Author manuscript; Available in PMC 2025 Feb 24. (PMC7617422; doi:10.1111/1755-0998.13749)
Supplement: Appendix S1 [file EMS203359-supplement-Appendix_S1.docx]

**Supplemental Information for:**

**First chromosome scale genomes of ithomiine butterflies (Nymphalidae: Ithomiini): comparative models for mimicry genetic studies**

Jérémy Gauthier, Joana Meier, Fabrice Legeai, Melanie McClure, Annabel Whibley, Anthony Bretaudeau, Hélène Boulain, Hugues Parrinello, Sam T. Mugford, Richard Durbin, Chenxi Zhou, Shane McCarthy, Christopher W. Wheat, Florence Piron-Prunier, Christelle Monsempes, Marie-Christine François, Paul Jay, Camille Noûs, Emma Persyn, Emmanuelle Jacquin-Joly, Camille Meslin, Nicolas Montagné, Claire Lemaitre and Marianne Elias

**Table of Contents:**

| **Supplementary Table 1** | Page 2 |
| --- | --- |
| **Supplementary Figure 1** | Page 3 |
| **Supplementary Figure 2** | Page 4 |
| **Supplementary Figure 3** | Page 5 |

**Supplementary Table 1.** Estimation of genome statistics including heterozygosity (Het), read error rate (err), genome size estimation and model fitting of the kmer distribution (fit).


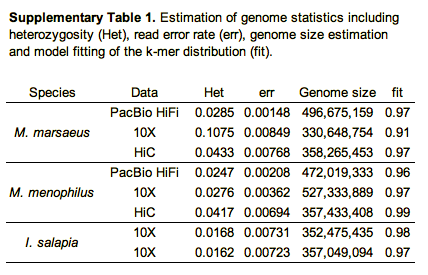


**Supplementary Figure 1.**k-mer profile and model fit as estimated with GenomeScopev.2.0 for each species and each sequencing library using a k-mer length of 31 bp.

**Supplementary Figure 2.** Hi-C contact map generated by SALSA2 for the chromosome scale genomes of *M. marsaeus* and *M. menophilus*.

**Supplementary Figure 3.** Transposable element content profiles of the three ithomiine species assemblies and *D. plexippus*.
